# Update to the AWED (Applying *Wolbachia* to Eliminate Dengue) trial study protocol: a cluster randomised controlled trial in Yogyakarta, Indonesia

**DOI:** 10.1186/s13063-020-04367-2

**Published:** 2020-05-25

**Authors:** Katherine L. Anders, Citra Indriani, Riris Andono Ahmad, Warsito Tantowijoyo, Eggi Arguni, Bekti Andari, Nicholas P. Jewell, Suzanne M. Dufault, Peter A. Ryan, Stephanie K. Tanamas, Edwige Rancès, Scott L. O’Neill, Cameron P. Simmons, Adi Utarini

**Affiliations:** 1 Institute of Vector Borne Disease, Monash University, 12 Innovation Walk, Melbourne, 3800 Victoria Australia; 2 Department of Biostatistics, Epidemiology and Population Health and Centre for Tropical Medicine, Faculty of Medicine, Universitas Gadjah Mada, Jl. Medika, Yogyakarta, 55281 Indonesia; 3 World Mosquito Program, Centre for Tropical Medicine, Faculty of Medicine, Universitas Gadjah Mada, Jl. Medika, Yogyakarta, 55281 Indonesia; 4 Department of Pediatrics and Centre for Tropical Medicine, Faculty of Medicine, Universitas Gadjah Mada, Jl. Medika, Yogyakarta, 55281 Indonesia; 5 Centre for Tropical Medicine, Faculty of Medicine, Universitas Gadjah Mada, Jl. Medika, Yogyakarta, 55281 Indonesia; 6 Centre for Statistical Methodology, London School of Hygiene and Tropical Medicine, Keppel St, London, WC1E 7HT UK; 7 School of Public Health, University of California, 2121 Berkeley Way, Berkeley, 94720-7360 CA USA; 8 Department of Health Policy and Management, and Centre for Tropical Medicine, Faculty of Medicine, Universitas Gadjah Mada, Jl. Medika, Yogyakarta, 55281 Indonesia

**Keywords:** *Wolbachia*, Dengue, Chikungunya, Zika, Vector-borne disease, Cluster randomised trial, Test-negative design, Indonesia

## Abstract

**Background:**

The AWED (Applying *Wolbachia* to Eliminate Dengue) trial is a parallel, two-arm, non-blinded cluster randomised controlled trial that is under way in Yogyakarta, Indonesia, with the aim of measuring the efficacy of *Wolbachia*-infected *Aedes aegypti* deployments in reducing dengue incidence in an endemic setting. Enrolment began in January 2018 and is ongoing. The original study protocol was published in April 2018. Here, we describe amendments that have been made to the study protocol since commencement of the trial.

**Methods:**

The key protocol amendments are (1) a revised study duration with planned end of participant enrolment in August 2020, (2) the addition of new secondary objectives (i) to estimate serotype-specific efficacy of the *Wolbachia* intervention and (ii) to compare *Ae. aegypti* abundance in intervention versus untreated clusters, (3) an additional exposure classification for the per-protocol analysis where the *Wolbachia* exposure index is calculated using only the cluster-level *Wolbachia* prevalence in the participant’s cluster of residence, (4) power re-estimation using a multinomial sampling method that better accounts for randomness in sampling, and (5) the addition of two trial stopping rules to address the potential for persistently low rates of virologically confirmed dengue case enrolment and *Wolbachia* contamination into untreated clusters. Additional minor changes to the protocol are also described.

**Discussion:**

The findings from this study will provide the first experimental evidence for the efficacy of *Wolbachia* in reducing dengue incidence. Enrolment in the trial will conclude this year (2020) and results will be reported shortly thereafter.

**Trial registration:**

ClinicalTrials.gov, identifier: NCT03055585. Registered on 14 February 2017. Last updated 22 March 2020.

## Update

This update relates to the study protocol for a cluster randomised controlled trial to evaluate the efficacy of *Wolbachia*-infected mosquito deployments to reduce dengue incidence in Yogyakarta, Indonesia: the Applying *Wolbachia* to Eliminate Dengue (AWED) trial. This update should be read in conjunction with the original protocol publication [1]. The trial registration record on ClinicalTrials.gov has been updated to reflect these protocol amendments.

## Study duration

A 12-month extension to the trial duration was approved by the independent data monitoring committee (IDMC) to account for lower-than-expected dengue incidence in Yogyakarta (and elsewhere in Indonesia) during the first year of the study period. Participant enrolment is now planned to conclude on 31 August 2020 unless early trial termination occurs because of *Wolbachia* contamination into untreated clusters or consistently low enrolment rates of virologically confirmed dengue (VCD) cases (see ‘Trial stopping rules’ below).

## Secondary endpoints

### DENV serotype-specific efficacy

The primary endpoint of the trial includes virologically confirmed dengue virus (DENV) infections of any (or unknown) serotype combined, based on the detection of DENV RNA in a pan-dengue reverse transcription polymerase chain reaction (RT-PCR) or detection of non-structural protein 1 (NS1) antigen. We now make explicit the use of a second serotype-specific RT-PCR to determine the infecting DENV serotype in samples positive in the pan-dengue PCR and to estimate serotype-specific efficacy of the *Wolbachia* intervention. In laboratory experiments, the degree to which *Wolbachia* reduces the DENV transmission potential of *Aedes aegypti* is dependent on the infecting virus serotype, and DENV1 transmission is least affected [2]. A secondary analysis will estimate the serotype-specific efficacy of *Wolbachia* deployments in reducing symptomatic dengue virus infection with a known infecting serotype, for each of the four serotypes in turn or as many as are detected in the study population. The same intention-to-treat and per-protocol analyses described for the primary endpoint will be used here. Case populations will be restricted to each of the DENV serotypes in turn, and the same control population will be used as for analysis of the primary endpoint.

### Zika and chikungunya

If at least 20 virologically confirmed Zika or chikungunya cases are detected, a secondary analysis will estimate the efficacy of *Wolbachia *deployments in reducing the incidence of symptomatic virologically confirmed Zika virus and chikungunya virus infection. No formal analysis will be undertaken if fewer than 20 virologically confirmed cases of Zika or chikungunya are detected. Only a descriptive analysis of the temporal and spatial distribution of cases will be carried out.

### Notified dengue cases

A proposed method for statistical analysis was added to the secondary endpoint of assessing the impact of *Wolbachia* deployment on routine dengue case notifications. An interrupted time series analysis of monthly dengue haemorrhagic fever (DHF) notifications by kelurahan, before and after *Wolbachia* releases, will be used to evaluate the impact of *Wolbachia* deployment on DHF case notifications. Methods will be developed and validated *a priori* to classify area-level *Wolbachia* exposure status in a way that aligns with the administrative (kelurahan) boundaries by which dengue cases are reported. A separate statistical analysis plan will be developed for this endpoint, and the results will be reported in a secondary publication subsequent to the publication of the main trial results.

### *Aedes* species abundance

The AWED trial provides an opportunity to explore whether fitness costs associated with *Wolbachia* infection of *Ae. aegypti* that have been identified in laboratory environments (e.g., fecundity and egg survivorship) manifest as a lower population size of adult mosquitoes in areas where *Wolbachia* is established versus untreated areas. A secondary endpoint was added to measure and compare the population size of adult mosquitoes in *Wolbachia*-treated versus untreated clusters using existing data from BG trap mosquito collections. Poisson regression will be used to test the null hypothesis of no difference in the abundance of *Ae. aegypti* and other species by treatment arm, incorporating BG trap as a random effect to account for the clustered sampling of mosquitoes by BG trap.

### Prevalence of arbovirus-infected *Aedes aegypti* mosquitoes

Assessing the impact of *Wolbachia* deployment on the prevalence of arbovirus-infected *Ae. aegypti* mosquitoes will no longer be carried out. Findings so far indicate that the overall prevalence of dengue virus-infected *Ae. aegypti* mosquitoes is too low for this secondary objective to be feasible within the resources available.

## Clinical sampling procedures

In a situation where a consenting participant has already had blood collected for clinical investigations on the day of enrolment, a second blood sample will not be collected for research purposes. Rather, the residual blood sample will be retained and used for study investigations. Two implications of this are noted: (1) the sample volume may be lower than the usual 3 mL, and (2) informed consent may be obtained from the participant after the clinical blood sampling has occurred (but before retrieval of the residual sample for use in the study). This amendment was made in response to a number of participants who were enrolled without a blood sample, and thus no classifiable diagnostic results to determine case/control status, due to their refusal to have two blood samples collected in one visit, one each for clinical diagnostic purposes and for our study.

## Diagnostic algorithm

**Fig. 1:**
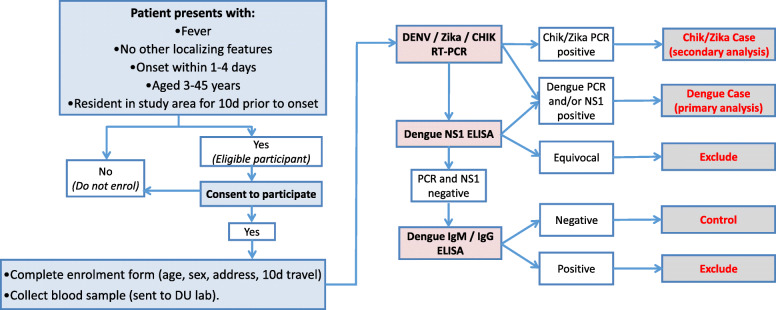
Flowchart of data and sample collection procedures and diagnostic algorithm. Blue boxes indicate participant recruitment and enrolment activities undertaken at Puskesmas clinics, including screening against inclusion/exclusion criteria, obtaining written informed consent, and collection of demographic and travel history data and a blood sample. Pink boxes indicate the laboratory diagnostic testing to be performed at the project laboratory (DU), the results of which (white boxes) will be used to classify participants as virologically confirmed dengue, Zika or chikungunya (Chik) cases or arbovirus-negative controls or excluded because of an inability to exclude arbovirus infection (grey boxes) according to the algorithm shown. This diagnostic algorithm is updated from the original version to indicate that all samples are tested by both reverse transcriptase polymerase chain reaction (RT-PCR) and dengue virus (DENV) non-structural protein 1 (NS1) enzyme-linked immunosorbent assay (ELISA). In the original algorithm, only samples that were PCR-negative for dengue, chikungunya and Zika were tested by NS1 ELISA

Previously, only samples that were PCR-negative for dengue, chikungunya and Zika were subsequently tested by DENV NS1 enzyme-linked immunosorbent assay (ELISA). All samples will now be tested by both RT-PCR and NS1 ELISA (Bio-Rad dengue NS1 Platelia ELISA, Bio-Rad Laboratories, Hercules, CA, USA) (Fig. 1). All samples positive for DENV in the triplex reverse transcription polymerase chain reaction (RT-PCR) will be tested in a serotype-specific RT-PCR to determine the infecting serotype.

## Per-protocol analysis

### Dataset for analysis

The same participant dataset will now be used for the intention-to-treat analysis and the per-protocol analysis (i.e., including all participants recruited after *Wolbachia* is considered established in the intervention area, defined as 1 month after completion of releases in the last cluster).

### Alternative exposure classification

An additional per-protocol analysis will be conducted in which the *Wolbachia* exposure index (WEI) is calculated using only the cluster-level *Wolbachia* prevalence in the participant’s cluster of residence (in the month of participant enrolment), ignoring the participant’s recent travel history. This recognises that dengue exposure risk may be higher at home versus other locations, rather than assuming an even distribution of exposure risk across daytime hours and locations visited.

### Revised statistical methods

A mixed-effects logistic regression model will be fitted, incorporating time as a random effect and with another random effect for cluster membership. Such models yield an estimate, and associated confidence interval, for the relative risk. The WEI strata will first be included as an ordinal covariate, and the slope of the WEI variable will be tested for a difference from zero. The WEI strata will additionally be included as a nominal (unordered) covariate to calculate stratum-specific incidence rate ratios (relative to the baseline 0–0.2 stratum). This will allow examination of a ‘dose response’ relationship. An additional benefit of transforming WEI to a categorical variable is that it avoids any assumption of linearity in the dose response relationship.

## Power calculations

Statistical power for the trial was re-estimated in January 2019 using a multinomial simulation method, with a range of sample sizes and effect sizes, as compared with the original power estimation which used a deterministic simulation of a range of effect sizes but with a fixed sample size. This revised method better accounts for randomness in sampling, including variability in the distribution of dengue cases and non-dengue febrile patients between clusters. This indicates that adequate power (≥80%) can be achieved with a substantially lower sample size than the 1000 dengue cases assumed for the original power simulation scenario. There is more than 80% power to detect a risk ratio (RR) of 0.5 (or smaller) with a sample of 400 VCD cases and 4 × 400 test-negative controls.

Additional simulations in September 2019 explored the potential impact on power if a number of untreated clusters are ‘lost’ to *Wolbachia* contamination. Given 400 enrolled VCD cases and a true effect size of 50% (RR = 0.5), contamination of three or six untreated clusters is expected to result in about 7% and about 14% loss of statistical power, respectively.

## Interim analysis

An interim analysis was originally planned for the mid-point of the study (i.e., after enrolment of 500 dengue cases from an initial target sample size of 1000). The re-estimation of statistical power described above indicates that the trial will be adequately powered even with a smaller sample size, and the threshold of 500 cases is unlikely to be reached. The multinomial sampling method used in the power re-estimation means that it is no longer necessary to re-calculate sample size using the observed inter-cluster distribution of participants, as originally stated. In November 2019, the IDMC advised that no interim analysis was required.

## Trial stopping rules

Additional criteria for early termination of the trial were introduced in October 2019 in response to increasing *Wolbachia* contamination in several untreated clusters. The first rule addresses the possibility that a power loss due to contamination may compromise the intention-to-treat analysis and would see the trial stop if five or more untreated clusters are classified as contaminated. A cluster will be defined as contaminated when the cluster-level *Wolbachia* frequency is more than 50% for two monthly monitoring events within a 6-month rolling window and more than 50% of the BG traps in the cluster have detected *Wolbachia* during those monitoring events.

The second rule addresses the potential for consistently low rates of VCD case enrolment to make continued recruitment until August 2020 futile in terms of increasing sample size (statistical power) and would see the trial stop if five or fewer VCD cases are enrolled in any 3-month rolling window (commencing 1 November 2019).

An assessment of both the *Wolbachia* monitoring results and accrual of VCD cases will be made each month. The final decision to terminate or modify the study continues to rest with the trial steering committee (TSC). The participant dataset for analysis will include all those cases enrolled up until the end of the calendar month in which the stopping rule was triggered, even if the date the TSC endorses the decision to stop the trial falls in the following month.

## Trial status

Recruitment into the trial began in January 2018 and is ongoing, and completion is expected by August 2020. The current approved protocol is version 5.1 approved by Universitas Gadjah Mada (UGM) ethics committee on 22 January 2020 and Monash University ethics committee on 31 January 2020. This update includes additional minor amendments made in protocol version 6.0, which was under institutional review board review in March 2020.

## Data Availability

The full trial protocol will be made publicly available at the time of publication of trial results. The datasets generated in this study will be made available upon reasonable request to the corresponding author.

## Abbreviations

AWED: Applying *Wolbachia* to Eliminate Dengue
DENV: Dengue virus
DHF: Dengue haemorrhagic fever
ELISA: Enzyme-linked immunosorbent assay
IDMC: Independent data monitoring committee
NS1: Non-structural protein 1
PCR: Polymerase chain reaction
RR: Risk ratio
RT-PCR: Reverse transcriptase polymerase chain reaction
TSC: Trial steering committee
VCD: Virologically confirmed dengue
WEI: *Wolbachia* exposure index
